# Dr. J. Glenn Ernest

**Published:** 1917-01

**Authors:** 


**Dr. J. Glenn Ernest,** Niagara 1894, of Chestnut Ridge, near Gasport, N. Y., died Dec. 5 at the Buffalo General Hospital.

